# Infection and coinfection by human papillomavirus, Epstein–Barr virus and Merkel cell polyomavirus in patients with squamous cell carcinoma of the larynx: a retrospective study

**DOI:** 10.7717/peerj.5834

**Published:** 2018-10-24

**Authors:** Jose Manuel Vazquez-Guillen, Gerardo C. Palacios-Saucedo, Lydia Guadalupe Rivera-Morales, Monica Valeria Alonzo-Morado, Saira Berenice Burciaga-Bernal, Maribel Montufar-Martinez, Rocio Ortiz-Lopez, Vianey Gonzalez-Villasana, Ana Carolina Martinez-Torres, Julio Cesar Serna-Hernandez, Silvia Judith Hernandez-Martinez, Edmundo Erbey Castelan-Maldonado, Angel Zavala-Pompa, Martha Socorro Montalvo-Bañuelos, Ricardo Garcia-Cabello, Ethel Corinthia Sanchez-Fresno, Cristina Rodriguez-Padilla

**Affiliations:** 1 Laboratorio de Inmunología y Virología, Facultad de Ciencias Biológicas, Universidad Autónoma de Nuevo León, Monterrey, Nuevo León, México; 2 División de Investigación, Departamentos de Otorrinolaringología y Anatomía Patológica, Unidad Médica de Alta Especialidad No. 25, Instituto Mexicano del Seguro Social, Monterrey, Nuevo León, México; 3 Centro de Investigación y Desarrollo en Ciencias de la Salud, Universidad Autónoma de Nuevo León, Monterrey, Nuevo León, México; 4 Escuela de Medicina y Ciencias de la Salud, Tecnologico de Monterrey, Monterrey, Nuevo Léon, México; 5 Departamento de Biología Celular y Genética, Facultad de Ciencias Biológicas, Universidad Autónoma de Nuevo León, Monterrey, Nuevo León, México; 6 Departamento de Foniatría, Hospital General de Zona No. 6, Instituto Mexicano del Seguro Social, Monterrey, Nuevo León, México

**Keywords:** Squamous cell carcinoma, Human papillomavirus, Laryngeal cancer, Epstein-Barr virus, Merkel cell Polyomavirus

## Abstract

**Background:**

Human papillomavirus (HPV) is recognized as an important risk factor for laryngeal carcinogenesis. Although HPV-16 and 18 have been strongly implicated, the presence of other high-risk HPV (HR-HPV) genotypes or the coinfection with Epstein-Barr virus (EBV) or Merkel cell polyomavirus (MCPV) may increase the risk, but their etiological association has not been definitively established.

**Methods:**

We characterized the genotype-specific HPV and the frequency of EBV and MCPV infections through the detection of their DNA in 195 laryngeal specimens of squamous cell carcinoma (SCC) histologically confirmed.

**Results:**

HPV DNA was detected in 93 (47.7%) specimens. HPV-11 was the most frequent with 68 cases (73.1%), and HPV-52 was the most frequently HR-HPV found with 51 cases, which corresponds to 54.8% of all HPV-positive specimens. EBV DNA was detected in 54 (27.7%) tumor tissue specimens of which 25 (46.3%) were in coinfection with HPV. MCPV DNA was detected only in 11 (5.6%) cases of which 5 (45.4%) were in coinfection with an HR-HPV. No association between the presence of DNA of the three examined viruses and the patient smoking habits, alcohol consumption, age, the keratinization status, differentiation grade, or localization of the tumor in the larynx were found.

**Discussion:**

HPV-52 was the most prevalent HR-HPV, which may suggest that this and other genotypes in addition to HPV-16 and 18 could be considered for prophylaxis. However, further studies including non-cancer larynx cases and the evaluation of other molecular markers and viral co-infection mechanisms are needed to determine the role of the different HR-HPV genotypes, EBV, and MCPV in the etiology of SCC of the larynx.

## Introduction

Laryngeal cancer is the second most common malignant neoplasm of the head and neck and squamous cell carcinoma (SCC) is the most frequent histological type with 90–95% of the cases (Akhter et al., 2011; Marioni et al., 2006). Tobacco and alcohol consumption are the major risk factors, however, is widely accepted that infection by viruses with oncogenic potential plays an important role in the neoplastic progression (Pytynia, Dahlstrom & Sturgis, 2014). Human papillomavirus (HPV) is one of the main viruses involved in the appearance of tumors since it constitutes a group of viruses with epithelial cell tropism and is associated with a great variety of cutaneous proliferations and mucosal squamous lesions (Benson et al., 2014; Kreimer et al., 2005). More than 200 HPV genotypes have been identified, which can be classified according to their oncogenic potential. Thus, genotypes 6, 11, 40, 42, 43, 44, 53, 54, 61, 72, 73, and 81 have a low potential to develop malignancy as they are associated with more benign conditions such as genital warts whereby they are considered as low-risk (LR-HPV) to cancer. Genotypes 16, 18, 31, 33, 35, 39, 45, 51, 52, 56, 58, and 59 are known as a high-risk HPV (HR-HPV) since they are directly associated with the development of cancer, including cervical, penile, anal, and throat (Fakhry & Gillison, 2006; Omland et al., 2014; Lajer & Von Buchwald, 2010). Several epidemiologic and biological studies have implicated the HR-HPV infection, mainly genotypes 16 and 18, as a potential etiological factor for the development of tumors in the upper aerodigestive tract but other well-defined oncogenic viruses, such as Epstein-Barr virus (EBV) or Merkel cell polyomavirus (MCPV), might have a role in the pathogenesis of this type of tumors (Gama et al., 2016; Sturgis, Wei & Spitz, 2004; De Oliveira et al., 2006) EBV belongs to the group of herpesviruses and its initial transmission is via the oropharynx, where it has been associated to the etiopathogenesis of an increasing number of cancers; however, the mechanism of the immunopathogenesis of the infection caused by this virus has not yet been elucidated (Hutt-Fletcher, 2017; Goldenberg et al., 2004; Muderris et al., 2013). Polyomaviruses are oncogenic viruses closely related to HPV that have the capacity to transform cellular morphology. MCPV was identified in 2008 by using next-generation sequencing technologies and it has been identified as the causal agent of a subset of Merkel cell carcinoma, a rare and aggressive neoplasia of the skin (Spurgeon & Lambert, 2013; Arora, Chang & Moore, 2012; Stakaityte et al., 2014; Liu et al., 2016; Mertz et al., 2010). Recently, some studies have reported the presence of MCPV in the airways, but its association with neoplasm in these anatomical sites has not been established (Wiel et al., 2009; Goh et al., 2009; Bialasiewicz et al., 2009). We conducted a study based on a third-level care hospital, whose coverage includes patients from all northeast Mexico, to determine the prevalence of the infection and coinfection by HPV, EBV, and MCPV in tumor tissues of laryngeal SCC cases.

## Material and Methods

### Samples and DNA isolation

Formalin-fixed paraffin embedded (FFPE) tissues from patients with histologically-confirmed SCC were included. Individual informed consent was not obtained as specimens were retrospectively collected. The National Committee of Investigation of the Instituto Mexicano del Seguro Social granted ethical approval to carry out the study (Reg. No. R-2014-785-055). Cases were diagnosed between 2012 and 2015 and belong to the Departamento de Otorrinolaringología y Anatomía Patológica at Unidad Médica de Alta Especialidad (UMAE) No. 25, a tertiary referral hospital of the Instituto Mexicano del Seguro Social (IMSS) located in northeastern Mexico. Information about clinical characteristics of patients such as age, sex, history of alcohol and tobacco use, tumor location, and histopathological diagnosis was obtained from their clinical records. It was considered with a history of chronic alcohol intake at a level of consumption that causes health risks: men >15 drinks/week or >5 drinks/day, women >8 drinks/week or >4 drinks/day) (Vokes, Agrawal & Seiwert, 2015). It was considered with a history of smoking when the clinical record referred consumption of 10 or more packages of cigarettes per year (Villalba-Caloca & Martinez-Heredero, 2004). All patient data were de-identified and anonymized prior to analysis. Laryngectomies or biopsies of all subsites of the larynx (subglottis, glottis, or supraglottis) were evaluated. Tumor and non-tumor areas of the FFPE blocks were delineated by two independent expert pathologists and representative tumor sections were carefully punched off and conducted to a DNA purification process using the NucleoSpin DNA FFPE (Macherey-Nagel, Düren, Germany).

### HPV genotyping and EBV and MCPV detection

The presence and specific genotyping of HPV DNA were evaluated using the INNO-LiPA HPV Genotyping Kit Extra II Amp (LiPA, Innogenetics, Gent, Belgium), a line probe assay designed for the identification of genotypes of the HPV by the detection of a 65 bp specific sequence in the L1 region of the HPV genome. This assay contains internal controls to monitor the sample quality and extraction efficiency. The EBV and MCPV detection were made after the HPV genotyping assays by independent reactions of real-time qPCR with hydrolysis probes that were designed based on previously published studies and using the TaqMan Universal PCR MasterMix (Applied Biosystems, Foster City CA, USA) in the Light Cycler (Roche) (Ryan et al., 2004; Varga et al., 2009). To detect DNA of EBV, a 69 bp DNA fragment between the 679–748 region of the viral LMP2 glycoprotein was amplified using the primers 5′-AGC TGT AAC TGT GGT TTC CAT GAC-3′ and 5′-GCC CCC TGG CGA AGA G-3′ and the probe 6FAM5′-CTG CTG CTA CTG GCT TTC GTC CTC TGG-3′TAMRA. The DNA of MCPV was detected using primers 5′-TAC AAG CAC TCC ACC AAA GC-3′ and 5′-CAG CAT GGC TAA GAT AAT CAG AAA C-3′ with the probe 6FAM5′-ACA ACA GAG AAA CTC CTG TTC CTA CT-3′TAMRA that amplify between the 1,514–1,606 region of the VP1 viral protein resulting in a 92 bp amplicon size. Synthetic DNA fragment (gBlocks Gene Fragment, IDT) that contains sequences from the amplicons of EBV and MCPV was used for amplification control and for the generation of standard curves to evaluate the efficiency and detection limits of both assays.

### Statistical analysis

The prevalence of HPV and their genotypes, EBV and MCPV was based on positive/negative detection of viral DNA in tumor tissues and was compared with the clinical and anatomopathological characteristics of patients by using the Pearson’s Chi-squared test, the Mann–Whitney *U* test or the Fisher’s exact test. The analysis was made using the IBM SPSS Statistics software (Version 20; SPSS. Inc., Chicago IL, USA).

## Results

A total of 215 specimens of FFPE tumor tissues with SCC diagnosis, clinical records, and complete histological reports were available, from which 20 were excluded because they tested negative for the internal controls of the HPV genotyping test. Therefore, the study sample included 195 specimens yielding valid results. No significant differences were found in the clinical data of excluded patients when compared with those of the studied group. None of the patients had a history of recurrent respiratory papillomatosis. Most patients belonged to the age group of sixty and over (74.9%); the majority were male (94.9%) and the majority were smokers (96.4%). The principal tumor location in the larynx was the glottis subsite (62.6%) and 67.2% of the studied specimens corresponded to laryngeal tumor biopsies and 32.8% to complete resections of the larynx (laryngectomies). Moderately differentiated grade with some keratinization was predominant (84.1%). Alcohol consumption was not a frequent feature (6.1%) (Table 1).

**Table 1:** Characteristics of 195 patients with laryngeal cancer classified according to the detection of human papillomavirus, Epstein Barr virus and Merkel cell polyomavirus in FFPE tumor tissue with SCC histologically-confirmed.

	**Total**	**Human papillomavirus**			**High-risk human papillomavirus**			**Epstein-Barr virus**			**Merkel cell polyomavirus**		
		**Positive**	**Negative**		**Positive**	**Negative**		**Positive**	**Negative**		**Positive**	**Negative**	
	**(*n* = 195)**	**(*n* = 93)**	**(*n* = 102)**	***p***	**(*n* = 67)**	**(*n* = 128)**	***p***	**(*n* = 54)**	**(*n* = 141)**	***p***	**(*n* = 11)**	**(*n* = 184)**	***p***
**Age (years)**	66 (35–89)	66 (44–84)	65.5 (35–89)	0.554	67 (49–84)	65 (35–89)	0.185	64.5 (40–89)	66 (35–87)	0.410	67 (45–81)	66 (35–89)	0.798
**≥60 years old**	146 (74.9%)	73 (50.0%)	73 (50.0%)	0.322	55 (37.7%)	91 (62.3%)	0.064	37 (25.3%)	109 (74.7%)	0.268	8 (5.5%)	138 (94.5%)	1.000
**Sex**				0.750						0.729			1.000
Male	185 (94.9%)	89 (48.1%)	96 (51.9%)		65 (35.1%)	120 (64.9%)		52 (28.1%)	133 (71.9%)		11 (5.9%)	174 (94.1%)	
Female	10 (5.1%)	4 (40.0%)	6 (60.0%)		2 (20.0%)	8 (80.0%)		2 (20.0%)	8 (80.0%)		–	10 (100%)	
**Surgical sample**				0.016			0.204			0.026			0.074
Biopsy	131 (67.2%)	55 (42.0%)	76 (58.0%)		41 (31.3%)	90 (68.7%)		30 (22.9%)	101 (77.1%)		10 (7.6%)	121 (92.4%)	
Laryngectomy	64 (32.8%)	38 (59.4%)	26 (40.6%)		26 (40.6%)	38 (59.4%)		24 (37.5%)	40 (62.5%)		1 (7.6%)	63 (98.4%)	
**Anatomical location**			0.596			0.931			0.082			0.084
Glottic	122 (62.6%)	54 (44.3%)	68 (55.7%)		42 (34.4%)	80 (65.6%)		27 (22.1%)	95 (77.9%)		9 (7.4%)	113 (92.6%)	
Subglottic	6 (3.1%)	3 (50.0%)	3 (50.0%)		2 (33.3%)	4 (66.7%)		1 (16.7%)	5 (83.3%)		1 (16.7%)	5 (83.3%)	
Supraglottic	24 (12.3%)	14 (58.3%)	10 (41.7%)		7 (29.2%)	17 (70.8%)		8 (33.3%)	16 (66.7%)		1 (4.2%)	23 (95.8%)	
Transglottic	43 (22.1%)	22 (51.2%)	21 (48.8%)		16 (37.2%)	27 (62.8%)		18 (41.9%)	25 (58.1%)		–	43 (100%)	
**Histopathological grading**			0.201			0.267			0.217			
HDI	23 (11.8%)	10 (43.5%)	13 (56.5%)		6 (26.1%)	17 (73.9%)		7 (30.4%)	16 (69.6%)		–	23 (100%)	
MDSK	164 (84.1%)	81 (49.4%)	83 (50.6%)		59 (36.0%)	105 (73.9%)		43 (26.2%)	121 (73.8%)		11 (6.7%)	153 (93.3%)	
MDNK	3 (1.5%)	2 (66.7%)	1 (33.3%)		2 (66.7%)	1 (33.3%)		1 (33.3%)	2 (66.7%)		–	3 (100%)	
HDK	2 (1.0%)	–	2 (100%)		–	2 (100%)		2 (100%)	-		–	2 (100%)	
HGSP	1 (0.5%)	–	1 (100%)		–	1 (100%)		–	1 (100%)		–	1 (100%)	
PDNK	2 (1.0%)	–	2 (100%)		–	2 (100%)		1 (50.0%)	1 (50.0%)		–	2 (100%)	
**Smoking**	188 (96.4%)	89 (47.3%)	99 (52.7%)	0.449	64 (34.0%)	124 (66.0%)	0.693	52 (27.7%)	136 (72.3%)	0.624	10 (5.3%)	178 (94.7%)	0.338
**Alcohol use**	12 (6.2%)	8 (66.7%)	4 (33.3%)	0.145	5 (41.7%)	7 (58.3%)	0.550	6 (50.0%)	6 (50.0%)	0.078	1 (5.3%)	11 (91.7%)	0.512

**Notes.**

Values are shown in absolute frequencies (percentage). Age is presented as a median (range).

HDI highly differentiated invasive MDSK moderately differentiated some keratinized MDNK moderately differentiated non-keratinized HDK highly differentiated keratinized HGSP high grade of sarcomatoid pattern PDNK poor grade of sarcomatoid pattern

**Table 2:** Clinical data and status of Epstein-Barr virus and Merkel cell polyomavirus detection in 67 laryngeal cancer patients positive for at least one high-risk human papillomavirus.

**Patient No.**	**Sex**	**Age**	**Surgical sample**	**Anatomical location**	**Histopathological grading**	**Smoking**	**Alcohol use**	**HR-HPV genotype**	**EBV**	**MCPV**
7	M	61	Laryngectomy	Subglottic	MDSK	Yes	Yes	11, 52	–	–
8	M	75	Biopsy	Glottic	MDSK	Yes	No	52	–	–
9	M	53	Laryngectomy	Transglottic	MDSK	Yes	No	16	–	–
10	M	61	Biopsy	Glottic	MDSK	Yes	No	52	–	–
13	M	61	Biopsy	Supraglottic	MDSK	Yes	No	52	–	–
15	M	63	Biopsy	Glottic	MDSK	Yes	No	16	–	+
28	M	71	Laryngectomy	Transglottic	MDSK	Yes	No	11, 52	–	–
29	M	68	Biopsy	Supraglottic	MDSK	Yes	No	11, 16	–	–
32	M	69	Biopsy	Glottic	MDSK	Yes	No	45	–	+
45	M	81	Biopsy	Glottic	MDSK	Yes	No	11, 52	–	+
46	M	67	Biopsy	Glottic	MDSK	Yes	No	11, 16	–	–
47	M	71	Laryngectomy	Transglottic	MDSK	Yes	No	6, 11, 52	–	–
49	M	63	Laryngectomy	Transglottic	MDSK	Yes	No	11, 52	–	–
50	M	68	Laryngectomy	Glottic	MDSK	No	Yes	11, 45, 52	–	–
51	M	83	Biopsy	Supraglottic	MDSK	Yes	No	6, 11, 52	–	–
52	M	66	Biopsy	Glottic	MDSK	Yes	No	16, 52	–	–
53	M	60	Laryngectomy	Transglottic	MDSK	Yes	No	11, 52	–	–
54	M	67	Biopsy	Glottic	HDI	Yes	No	45	–	–
55	M	69	Biopsy	Glottic	MDSK	Yes	No	6, 11, 52	–	–
56	M	60	Laryngectomy	Glottic	MDSK	Yes	No	11, 45	–	–
57	F	72	Biopsy	Glottic	HDI	Yes	No	11, 52	+	–
58	M	72	Laryngectomy	Glottic	MDSK	Yes	No	11, 52	–	–
59	M	51	Laryngectomy	Transglottic	MDSK	Yes	No	52	+	–
60	M	61	Biopsy	Glottic	MDSK	Yes	No	11, 52	–	–
61	M	78	Biopsy	Glottic	MDSK	Yes	Yes	11, 16	–	–
62	M	61	Laryngectomy	Transglottic	MDSK	Yes	No	6, 11, 52	–	–
63	M	56	Laryngectomy	Subglottic	MDSK	Yes	No	11, 31, 52, 54	+	–
64	M	62	Biopsy	Glottic	MDSK	Yes	No	11, 52	–	–
65	M	79	Biopsy	Glottic	MDSK	Yes	No	11, 52	+	–
66	M	84	Laryngectomy	Glottic	MDSK	Yes	No	6, 11, 52	–	–
67	M	55	Biopsy	Glottic	MDSK	Yes	No	6, 11, 52	+	–
68	M	65	Laryngectomy	Transglottic	MDSK	Yes	Yes	11, 16, 52	+	–
69	M	58	Laryngectomy	Transglottic	MDSK	Yes	No	11, 52	–	–
70	M	72	Laryngectomy	Transglottic	MDSK	No	No	11, 52	+	–
71	M	74	Laryngectomy	Transglottic	MDSK	Yes	No	11, 31, 52, 54	–	–
72	M	71	Laryngectomy	Transglottic	MDSK	Yes	No	11, 52	–	–
73	M	67	Laryngectomy	Glottic	MDSK	Yes	No	52	–	–
74	M	72	Laryngectomy	Supraglottic	MDSK	Yes	No	11, 31, 52, 54	+	–
75	M	84	Laryngectomy	Glottic	MDSK	Yes	No	11, 26, 31, 52, 54	+	–
79	M	70	Biopsy	Glottic	MDNK	Yes	No	11, 31, 52, 54	–	–
80	M	49	Biopsy	Glottic	HDI	Yes	No	11, 52	–	–
81	M	72	Laryngectomy	Glottic	MDSK	Yes	No	11, 31, 52, 54	–	–
84	M	56	Biopsy	Glottic	MDSK	Yes	No	6, 11, 16, 52	–	–
85	M	79	Biopsy	Supraglottic	MDSK	Yes	No	11, 52	–	–
86	M	63	Biopsy	Glottic	MDSK	Yes	No	11, 52	–	–
88	M	68	Laryngectomy	Transglottic	MDSK	Yes	No	6, 11, 52	+	–
89	M	60	Biopsy	Glottic	MDSK	Yes	No	11, 52	–	–
90	M	62	Laryngectomy	Transglottic	MDSK	Yes	No	11, 31, 52	+	–
91	M	75	Biopsy	Glottic	MDSK	Yes	No	11, 52	–	–
92	M	76	Laryngectomy	Transglottic	MDSK	Yes	No	11, 31, 52	–	–
97	M	49	Laryngectomy	Glottic	MDSK	Yes	No	52	–	–
99	M	67	Biopsy	Supraglottic	MDSK	Yes	No	52	–	–
102	M	63	Biopsy	Supraglottic	HDI	Yes	No	31, 52, 54	–	–
113	M	76	Biopsy	Glottic	MDSK	Yes	No	52	–	–
114	M	71	Biopsy	Glottic	MDSK	Yes	No	52	–	–
119	M	49	Biopsy	Transglottic	MDSK	Yes	No	45	+	–
120	M	77	Biopsy	Glottic	MDSK	Yes	No	45	+	–
124	F	75	Biopsy	Glottic	MDSK	Yes	No	45	–	–
133	M	63	Biopsy	Glottic	HDI	Yes	No	45	–	–
139	M	69	Biopsy	Glottic	MDSK	Yes	Yes	52	+	–
141	M	58	Biopsy	Glottic	MDSK	Yes	No	52	–	+
144	M	65	Biopsy	Glottic	HDI	Yes	No	31, 52	–	–
148	M	62	Biopsy	Glottic	MDSK	Yes	No	52	+	–
149	M	75	Biopsy	Glottic	MDSK	No	No	45	+	+
180	M	54	Biopsy	Glottic	MDSK	Yes	No	11, 16	–	–
184	M	64	Biopsy	Glottic	MDSK	Yes	No	11, 16, 40, 82	–	–
195	M	59	Biopsy	Glottic	MDNK	Yes	No	16	–	–

**Notes.**

HR-HPV high-risk human papillomavirus EBV Epstein-Barr virus MCPV Merkel cell polyomavirus

Sex is indicated as M, male or F, female.

HDI highly differentiated invasive MDNK moderately differentiated non-keratinized MDSK moderately differentiated some keratinized

**Table 3:** Clinical data and status of Epstein-Barr virus and Merkel cell polyomavirus detection in 26 laryngeal cancer patients positive for Low-Risk Human Papillomavirus.

**Patient No.**	**Sex**	**Age**	**Surgical sample**	**Anatomical location**	**Histopathological grading**	**Smoking**	**Alcohol use**	**LR-HPV genotype**	**EBV**	**MCPV**
42	M	79	Biopsy	Supraglottic	MDSK	Yes	No	11	–	–
48	M	80	Biopsy	Supraglottic	MDSK	Yes	No	6, 11	–	–
76	M	64	Laryngectomy	Glottic	MDSK	Yes	No	11	+	–
77	M	44	Laryngectomy	Transglottic	MDSK	Yes	No	11	–	–
78	M	62	Biopsy	Supraglottic	MDSK	Yes	No	11	+	–
87	M	65	Laryngectomy	Glottic	MDSK	Yes	No	6, 11	+	–
98	M	49	Laryngectomy	Glottic	MDSK	Yes	No	11	–	–
116	F	61	Biopsy	Glottic	MDSK	Yes	No	11	–	–
125	M	49	Laryngectomy	Transglottic	HDI	Yes	No	11	+	–
130	M	49	Laryngectomy	Transglottic	HDI	Yes	No	11	–	–
152	M	52	Biopsy	Glottic	MDSK	Yes	No	11	–	–
156	M	66	Biopsy	Supraglottic	HDI	Yes	Yes	11	+	–
157	M	66	Biopsy	Glottic	MDSK	Yes	No	11	–	–
160	M	80	Biopsy	Glottic	MDSK	No	No	11	–	–
165	M	67	Biopsy	Supraglottic	MDSK	Yes	No	11	–	–
168	F	54	Biopsy	Glottic	MDSK	Yes	No	11	–	–
169	M	49	Laryngectomy	Transglottic	HDI	Yes	No	11	+	–
171	M	76	Biopsy	Supraglottic	MDSK	Yes	No	11	–	–
173	M	71	Laryngectomy	Transglottic	MDSK	Yes	No	11	+	–
177	M	70	Laryngectomy	Glottic	MDSK	Yes	No	11	–	–
178	M	58	Laryngectomy	Subglottic	MDSK	Yes	No	11	–	–
181	M	65	Laryngectomy	Glottic	MDSK	Yes	Yes	11	+	–
183	M	66	Biopsy	Glottic	MDSK	Yes	No	11	–	–
185	M	67	Biopsy	Supraglottic	MDSK	Yes	No	11	–	–
191	M	64	Laryngectomy	Transglottic	MDSK	Yes	No	11	–	–
194	M	67	Biopsy	Glottic	MDSK	Yes	Yes	11	+	–

**Notes.**

LR-HPV low-risk human papillomavirus EBV Epstein-Barr virus MCPV Merkel cell polyomavirus

Sex is indicated as M, male or F, female.

HDI highly differentiated invasive MDNK moderately differentiated non-keratinized MDSK moderately differentiated some keratinized

HPV DNA was detected in 93 (47.7%) tumor tissue specimens and ten different genotypes were identified, six HR-HPV and four LR-HPV. DNA of at least one HR-HPV was found in 67 (72% of the HPV-positive tumors), and 55 (82.1%) of these were detected in samples from patients of sixty years of age or older (Table 2). HPV-52 was the second most frequently detected genotype with 51 cases corresponding to 54.8% of the HPV-positives specimens, 12 (23.5%) as a single infection and 39 (76.5%) combined with other genotypes. HPV-16 was detected in 11 (11.8%) cases, of which three (27.3%) were a single detection and eight (72.7%) were a combination with other. DNA of HPV-31 was found in 10 cases (10.75%) and always combined with HPV-52 and other genotypes. HPV-45 was detected in nine (9.7%), 2 (22.2%) in combination and 7 (77.8%) as a single detection. DNA of HPV-26 and HPV-82 were detected only once (1.1%) and combined with other genotypes. HPV-11 was the most frequently detected with 68 (73.1%) cases of which, 24 (35.3%) were found as a single genotype and 44 (64.7%) were in co-infection with other genotypes. HPV-6 was detected in 10 cases (10.7%) and always in combination with others. HPV-40 and HPV-54 were found in 7 (7.5%) and 1 (1.1%) cases, respectively, and always in combination with other genotypes. Characteristics of 26 specimens positive for LR-HPV and without presence of HR-HPV are described in Table 3. EBV DNA was detected in 54 (27.7%) tumor tissue specimens of which, 25 (46.3%) were combined with HPV DNA, 16 (29.6%) with HR-HPV detection and three (5.5%) with MCPV DNA. Only 11 (5.6%) cases were positive for MCPV DNA and of these, five (45.4%) were positive for HR-HPV DNA detection. Only one specimen was positive for DNA of the three evaluated viruses and according to their clinical record, this patient did not consume tobacco and alcohol. Nonsignificant differences (*p* > 0.05) were observed between the presence of viral DNA of the three viruses evaluated and the patient smoking habits, alcohol consumption, age, the keratinization status, differentiation grade and subsite localization of the tumor in the larynx.

A comparison was made between some of the variables we evaluated. Hence, smoking was not associated with a higher probability of infection by one or multiple HPV genotypes or with the presence of EBV or MCPV infection. This finding could be explained by the fact that most of the patients whose samples were evaluated had a smoking history (188/195, 96.4%). The use of alcohol was also not associated with the detection of one or multiple HPV genotypes or with the presence of EBV or MCPV, although alcohol use was present only in 12 out of the 195 samples evaluated (6.2%). Having an age ≥60 years was associated with a higher frequency of detecting multiple HPV genotypes, 41 out of the 146 patients with this age (28.1%) had a multiple HPV genotype infection, whereas only 6 out of the 49 patients with an age <60 years (12.2%) had this kind of multiple infection (*p* = 0.033 by the Fisher’s Exact Test). Thus, the median age of patients with multiple HPV genotype infection was 68 years (range 49–84) and that of patients with infection by a single HPV genotype was 65 years (35-89) (*p* = 0.055 by the Mann–Whitney *U* Test). Having an infection by multiple HPV genotypes was also associated with a higher probability of detecting HR-HPV genotypes, in 45 out of the 47 patients with multiple HPV infection (95.7%) we detected one or more HR-HPV, whereas only in 22 out of the 148 patients with non-multiple HPV infection (14.9%) these HR-HPV genotypes were detected (*p* < 0.001 by Fisker’s Exact Test). The detection of multiple HPV genotypes was not associated with the identification of EBV. Furthermore, none of the specific HPV genotypes, either as a single or multiple infection, was associated with the detection of EBV.

## Discussion

Laryngeal cancer is the second most frequent head and neck cancer, with SCC the most common histological type. HPV infection is a strongly associated and numerous studies have shown that infection with HR-HPV genotypes represents an associated factor for its development (Gillison et al., 2000; Vokes, Agrawal & Seiwert, 2015). In addition, other viruses with oncogenic potential such as EBV and MCPV that have been detected in the nasopharyngeal tract, tonsils, salivary glands, and lungs may play an important role (Baez et al., 2015; Pezzuto et al., 2015; Gupta et al., 2016). At present, there are insufficient reports describing the epidemiology of viral coinfections in tumors of the larynx, so studies in this regard could contribute to clarify their association with the carcinogenic progression and to facilitate preventive strategies (Bansal, Singh & Rai, 2016). A total of 195 specimens of laryngeal tumor tissue were included in the present study. The epidemiological characteristics of the patients from whom these specimens were obtained are consistent with those described in the literature for this condition. The median age of the patients was 66 years and most of them were men, agreeing with several studies that have found a higher proportion of cases of laryngeal cancer in male patients older than 60 years (Hernandez et al., 2014; Urban, Corry & Rischin, 2014). The low number of female specimens precluded comparisons on the effects of sex. Epidemiological evidence shows smoking as an important risk factor in the pathogenesis of laryngeal tumors. This habit was a frequent finding among the patients included in the present study. Although alcoholism is also described as a risk condition for this type of tumors, its frequency was low in our study (Anttila & Boffetta, 2014; Simard, Torre & Jemal, 2014). The most frequent location of SCC was the glottis, which has been described as the most common site for laryngeal tumors (Armada & Tamez, 2005). A relationship between tobacco consumption and glottic localization of laryngeal tumors has been described so that the high frequency of smoking in our sample could be related to the high prevalence of glottic tumors that we found (Anttila & Boffetta, 2014).

The prevalence of 47.7% of HPV positive cases in the present study is similar to a number of studies reporting a prevalence of 40 to 50%; however, we did not observe a significant association between factors such as age, gender, the anatomical location of the tumor, and the histopathological damage with the presence of HPV DNA. The only difference found related to HPV positivity was between specimens obtained from biopsy and those from laryngectomy (Table 1). From the anatomopathological point of view, the amount of tissue available for microscopic examination is greater in tissue obtained by laryngectomy than by incisional biopsy. Of the total HPV positive cases, 72% had at least one HR-HPV genotype, but it was not possible to establish a significant association (*p* < 0.05) with the patient’s medical history. It is generally accepted that genotypes 16 and 18 of HPV are responsible for most laryngeal tumors since they are the genotypes most frequently identified in prevalence studies; however, in the present study the prevalence of HPV-52 was higher than those for any other high-risk HPV evaluated, which may suggest that HPV-52 could also have a role in laryngeal cancer (Pytynia, Dahlstrom & Sturgis, 2014; Hernandez et al., 2014; Huang et al., 1997).

On the other hand, several studies have evaluated the prevalence of EBV infection in laryngeal SCC reporting variable results that in general do not exceed 50% of the studied patients. Muderris et al. identified EBV in 10 (40%) of 25 patients (Muderris et al., 2013); Gök et al. reported the presence of this virus in 11 (50%) cases of a study carried out on 22 FFPE samples (Gök et al., 2003); and Vlachtsis et al. demonstrated the presence of EBV DNA in 39 (43.3%) of 90 patients (Vlachtsis et al., 2005). In the present study, EBV DNA was found in 54 (27.7%) of the 195 specimens of laryngeal tumors evaluated. This is a relatively low prevalence. However, Atula et al., and De Oliveira et al. did not identify EBV DNA in 79 (0%) and 110 (0%) specimens evaluated, respectively (De Oliveira et al., 2006; Atula et al., 1997). It is possible that this wide variability in the detection of this virus is due to the number of specimens evaluated and not to the detection method used since all these studies were performed using molecular detection techniques. As in these previously mentioned studies, we did not find a significant association between the presence of EBV DNA and the location and differentiation degree of the larynx tumor. Our results, however, must be considered with caution because it has been estimated that EBV latently infected B cells may be found in circulating cells in healthy individuals and false-positive results must be considered when PCR-based methods are used for EBV detection (De Oliveira et al., 2006). Furthermore, it is important to consider differences in sensitivity of EBV DNA detection as some studies have reported a higher sensitivity targeting BamHI-W than LMP2 region, which is the region we targeted (Sanosyan et al., 2017). Other methods, such as the detection of EBV-RNA transcripts in tissues can provide broader understanding of transcriptional activity in latent and lytic EBV infection, which could yield new insights on its pathogenic role (Greijer et al., 2017).

Several studies have reported the presence of MCPV in the respiratory tract but its association with neoplasia in this anatomical site has not been established (Goh et al., 2009; Bialasiewicz et al., 2009). We found 11 positive cases (5.6%) for this virus of the 195 specimens evaluated. This low prevalence is in accordance with the studies by Goh et al. (Goh et al., 2009), who reported the presence of MCPV DNA in 27 (4.3%) of 635 samples of nasopharyngeal aspirates from patients with a respiratory disease, and by Bialasiewicz et al. (2009), who identified seven (1.3%) MCPV DNA positive cases of 526 samples of nasopharyngeal aspirates and bronchoalveolar lavages of patients with respiratory viral infections. We did not evaluate the viral load or the number of copies of MCPV DNA although it is probable that they could be important to make appropriate interpretations of the participation of this virus in the development of tumors in the larynx; however, in the study carried out by Yahyapour et al. (2016), no significant differences regarding the number of copies of MCPV DNA were found between samples with SCC of esophagus and non-cancerous esophageal specimens.

HPV DNA was detected in almost half of the samples evaluated. Our finding of HPV-52 as the most frequent HR-HPV suggests that this genotype may be playing a role in laryngeal cancer etiology and that could be considered in prevention strategies. Although commercially available prophylactic vaccines against HPV were designed to prevent virus-induced cervix cancer, they could be an important strategy to prevent cancer in other sites since there is no a theoretical reason to expect that these vaccines would fail to induce protection in other anatomical localizations such as oral cavity, pharynx, and larynx (Testi et al., 2016). Though most widely used vaccines are the bivalent vaccine against HPV-16 and 18, and the tetra-valent vaccine for genotypes 6, 11, 16 and 18, our results show that other genotypes could be considered for prophylaxis strategies. In relation to this, the nine-valent vaccine also includes HPV-31, 33, 45, 52, and 58 (Kash et al., 2015; Panatto et al., 2015).

The interaction of multiple oncogenic viruses may be an important risk factor in the development of neoplasia that must be considered. Some studies have evaluated the prevalence of coinfections with HPV and other viruses. Thus, Drop et al. (2017) proposed that coinfections with HR-HPV and EBV may impact in the initiation and/or progression of tumors, and reported 28 (34%) cases with HPV and EBV when analyzing coinfections in fresh frozen tumor tissue (FFTT) samples obtained from patients with laryngeal, oropharyngeal or oral cancer. In our study, EBV DNA was detected in 54 (27.7%) samples, of which 25 (46.3%) were in coinfection with HPV, 16 (29.6%) of which were HR-HPV. Coinfections of HPV or EBV with other viruses have also been evaluated. Polz-Gruszka et al., identified coinfections with HPV, EBV and/or Cytomegalovirus at a low prevalence (<10%) in 80 FFTT samples from patients with oral SCC (Polz-Gruszka et al., 2015). We found a low prevalence of coinfections with MCPV and only one case of coinfection with the three viruses evaluated. However, Drop et al., reported a prevalence of 22% and 23.2% of BK polyomavirus (BKPV) with HPV and EBV, respectively (Drop et al., 2017). The role of these viruses in the pathogenesis of laryngeal carcinoma is still unclear, so further studies on the mechanisms of viral co-infection are necessary, including studies evaluating other molecular factors related to transformation and studies with larger numbers of fresh tissue samples, but especially studies including cancer-free controls are necessary to determine the true etiological role of these viruses in the neoplastic transformation.

## Conclusions

HPV infection was detected in 47.7% of the laryngeal tissue specimens of SCC examined, being HPV-11 the most frequent and HPV-52 the most common HR-HPV, which highlights the importance to consider this and other high-risk genotypes for prophylactic strategies. On the other hand, 27.7% of laryngeal tumor tissues were positive for EBV and only 5.6% of these tissues were positive for MCPV. Further studies including non-cancer larynx cases and the evaluation of other molecular markers and viral co-infection mechanisms are needed to clarify the etiologic role of these viruses in laryngeal SCC.

##  Supplemental Information

10.7717/peerj.5834/supp-1 Supplemental Information 1 Raw data used to generate statistics

## Additional Information and Declarations

### Data Availability

The following information was supplied regarding data availability: The raw data are provided in a Supplemental File.
